# The role of extracellular vesicles in renal fibrosis

**DOI:** 10.1038/s41419-019-1605-2

**Published:** 2019-05-08

**Authors:** H. Jing, S. Tang, S. Lin, M. Liao, H. Chen, J. Zhou

**Affiliations:** 1 Zhuhai Campus of Zunyi Medical University, The first People’s Hospital of Foshan, Golden Coast, Jinwan District, Zhuhai City, Guangdong Province China; 2 The first People’s Hospital of Foshan, 81 Lingnan Avenue, Chancheng District, Foshan City, Guangdong Province China

**Keywords:** Diagnostic markers, End-stage renal disease

## Abstract

As a particularly important mediator of intercellular communication, extracellular vesicles (EVs) have been proved to be extensively involved in various system diseases over the past two decades, including in renal diseases. As is well-known, renal fibrosis is the common pathological process of any ongoing renal disease or adaptive repair of kidney injury based on current knowledge. Although much work has been performed focusing on EVs in various renal diseases, the role of EVs in renal fibrosis has not been described in detail and summarized. In this review, we provide a brief overview of the definition, classification and biological process of EVs. Then, the potential mechanisms of EVs in renal fibrosis are illustrated. Lastly, recent advances in EVs and the implications of EVs for diagnosis and therapy in renal fibrosis disease are introduced. We look forward to a more comprehensive understanding of EVs in renal fibrosis, which could be a boon to patients with renal fibrosis disease.

## Facts

- EVs carry proteins, lipids, and RNAs that deliver molecular information between cell communication, thereby affecting the physiological and pathological states of receptor cells.
- Since the lipid bimolecular structure can be isolated from the extracellular environment, the content of EVs can be used as a tool for the diagnosis of renal fibrosis.
- EVs mediates the communication between different renal cells and is associated with the progression of renal fibrosis.

## Open questions

- How is EV localized and transported to target cells during renal cell–cell communication?
- What are the main components that mediate the function of EVs?
- Do we focus on whether RNA in EVs is reasonable in the process of renal fibrosis?

## Introduction

The main physiological function of extracellular vesicles (EVs) was believed to be the excretion of cell waste in earlier years^1^. However, we know today that the role of EVs is more than eliminating unneeded compounds nowadays. A mountain of reliable evidence has shown that EVs are important vehicles of intercellular communication^2–4^. EVs carry proteins, lipids, and RNAs that deliver molecular information between cell communication, thereby affecting the physiological and pathological states of receptor cells^5,6^. We often use EVs as the umbrella term for all types of vesicles in extracellular fluid, and they are generally classified into three categories (exosomes, microvesicles, and apoptotic bodies) based on their size and biological origin. With the evolution of the study of EVs, they have been found to be exist in many different biological fluids in addition blood, such as latex, saliva, urine, and cerebrospinal fluid^7^. This discovery laid the foundation for the clinical application of EVs. For example, the extraction of EVs from body fluids can act as biomarkers for renal diseases^8–10^. Furthermore, metabolic EV contents can serve as the response of cells to external pressures, including hypothermia, hypoxia, oxidative stress, and infectious pathogens. These facts suggest that EVs are involved in intracellular and intercellular signaling transmission and mediated a complex and multifarious mechanism to maintain physiological balance^11^.

In recent years, mounting evidence of the potential role of EVs in human diseases were unearthed^12–16^, and renal disease is no exception^17–20^. As is known, renal fibrosis is a common ultimate outcome of almost all chronic and progressive kidney diseases at the histological level. Therefore, it could be very meaningful to clarify the role of EVs in renal fibrosis. As people become more familiar with EVs, their value has been increasingly explored. Researchers have found that the contents of the EVs can be used as a diagnostic tool in renal fibrosis because the lipid bimolecular structure can be isolated from the extracellular environment^5,21^. Recently, the treatment of chronic kidney disease (CKD) to improve the degree of renal fibrosis by blocking EVs has been received great attention and has great prospects. Thus, EVs could be used as a diagnostic tool and for drug delivery^22–24^.

As mentioned above, although the pathophysiological roles for EVs have begun to be recognized in renal diseases, including DN, IgA nephropathy (IgAN) and so on^25,26^, there are still no reviews to specify the pathophysiological role of EVs in renal fibrosis. Therefore, we first briefly introduce EVs and then describe in detail how EVs participate in the renal fibrosis process at the cellular and molecular levels. In addition, the clinical application of EVs in renal fibrotic diseases, including their diagnostic value and therapeutic potential, is described.

## EVs

**Fig. 1:**
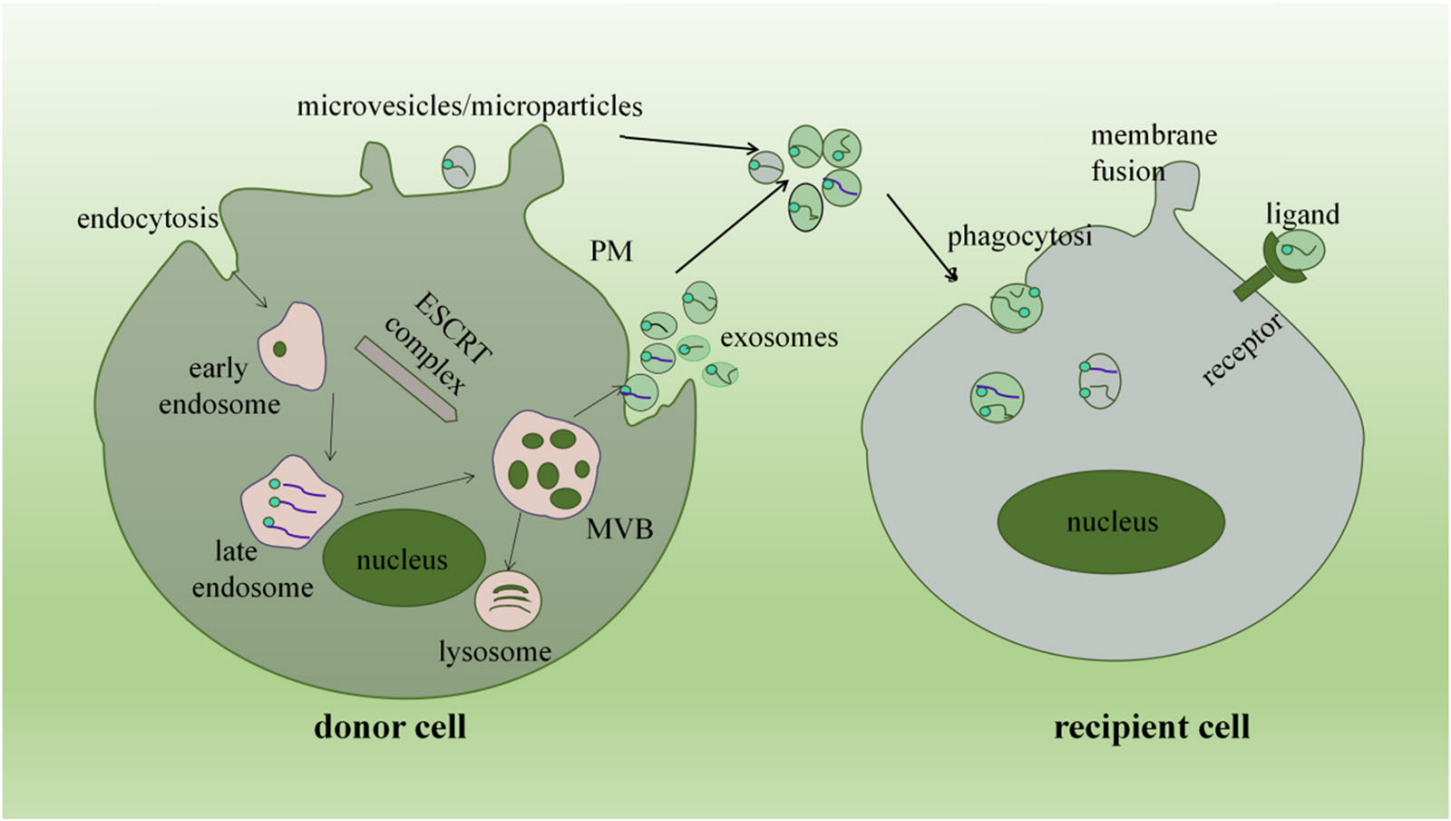
Secretion and transport of exosomes and microvesicles. The biological origin of exosomes can be divided into three stages: endosomes, multivesicular bodies (MVBs), and exosomes. Early endosomes are formed by the buds of the membrane formed by endocytosis. Subsequently, early endosomal vesicle membranes bud inward to form intraluminal vesicles, and then they selectively sort the proteins and lipids in the cytoplasm to form the late endosomes, namely MVBs. All these events are regulated by a so-called ESCRT complex (the endosome complex required for transport). However, the formation of MVB may also occur in ways that do not depend on ESCRT, such as through the tetraspanin CD63, the lipid metabolism enzymes sphingomyelinase, and phospholipase D2. The assembled MVBs can fuse with either lysosomes for cargo degradation or bind to the plasma membrane (PM) to release vesicles. Once released extracellularly, these vesicles are called exosomes (Fig. 1). MVs, sometimes called microparticles (MPs), are produced directly through outward budding and shed from the plasma membrane. The process is initially triggered by an increase in intracellular calcium ions in the cells, which activate caloproteinase, separating the membrane protein from the cytoskeleton inside the cells. EVs is then transported to the target cells, and their contents can be transmitted to cytoplasm through either fuse with the plasma membrane of the target cell or are internalized into the endocytic network, or by binding to the target cell receptor via EVs surface ligand, thereby modifying the physiological state of the recipient cell

EVs are a heterogeneous family of membrane-bound vesicles released from the surface of cells originating from the endosome or plasma membrane^27^. From disposing of cell waste to being an important carrier^28^, the recognition of EVs is becoming increasingly mature. According to their size, biological origin and secretion mechanisms, three basic types of generalized EVs have been proposed, including exosomes, microvesicles (MVs), and apoptotic bodies^29^ (Table 1). In fact, the narrow sense EVs only refers to first two types. Therefore, the present review focuses mainly on exosomes and MVs. Exosomes are the most characteristic of EV subtypes and are produced by endosomal pathways^30^. MVs, sometimes called microparticles (MPs), are produced directly through outward budding and shed from the plasma membrane^27^ (Fig. 1). Apoptotic bodies are formed at the late stage of cell contraction/collapse, after the externalization of phosphatidylserine, the increase of cell membrane permeability and nuclear fragmentation^31^. Several abbreviations of EVs in this review, including EVs, MVs, and MPs. EVs are the collective names of several types of vesicles. Both MVs and MPs refer to the abbreviation of EVs that sprout directly from the plasma membrane.

**Table 1:** Main differences among three common EVs

	Exosomes	Microvesicles/Microparticles	Apoptotic vesicles
Size	50–100 nm	100–1000 nm	100–5000 nm
Origin	Endosomal pathway (viable cells)	Budding of the PM (viable cells)	Apoptotic cells (at apoptotic stage)
Composition	mRNAs, miRNAs, other ncRNAs, proteins, lipids	mRNAs, miRNAs, other ncRNAs, proteins, lipids	DNA, rRNAs, organelles, proteins, lipids
Release	Formation by endosomal pathway, budding after fusion of MVBs and plasma membranes	Cell skeleton reorganization, outward budding of the PM	Outward blebbing of apoptotic cell membrane
Markers	Lamp1,TSG101, membrane protein CD63	Membrane protein CD40	A large amount of phosphatidylserine

### Methods of isolation

In recent years, EVs have been extensively explored in various diseases. Therefore, the isolation and purification of EVs has become a research hotspot. In various studies on the role of EVs in renal fibrosis, the most common EVs extract is urine^32,33^. At present, there is no general method to isolate and purify EVs for all studies. In the current study, commonly used methods for isolation EVs include ultracentrifugation, immunoisolation, and ultrafiltration^34,35^. It is well known that ultracentrifugation is a classical method and gold standard for obtaining and separating EVs. Immunoisolation is another method for isolation and purification of EVs. It uses magnetic beads coated with antibodies to recognize certain proteins on the lipid bilayer membranes of EVs, thus separating them from other substances. Ultrafiltration is a simple and easy method to isolate EVs depending on size. The advantages and disadvantages of the three methods are shown in Table 2. Nowadays, most researchers use one or more other techniques after the main steps, such as washing in EV-free buffer, ultrafiltration, and further purification by density gradient^36^. Although these methods can be used for the isolation of EVs, the purification of exosomes remains a great challenge, especially in the isolation of EVs from liposomes, proteins and RNA contamination.

**Fig. 2:**
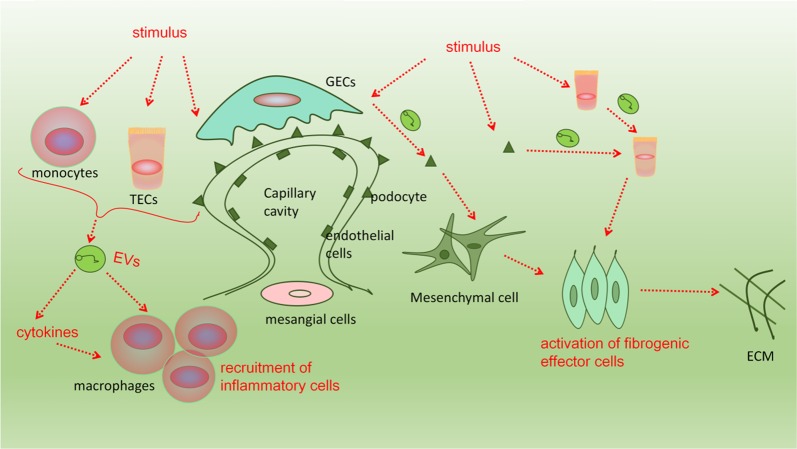
EVs in cellular mechanisms of renal fibrosis. Various stimulants act on cells in the kidney. EVs secreted by damaged kidney cells increases and their contents also change. EVs induce the release of cytokines and promote the aggregation of inflammatory cells. In addition, EVs secreted by damaged kidney cells can be transferred to other normal kidney cells and change the phenotype of normal kidney cells and the activation of fibroblasts, which creates a vicious cycle and thereby promotes renal fibrosis

**Fig. 3:**
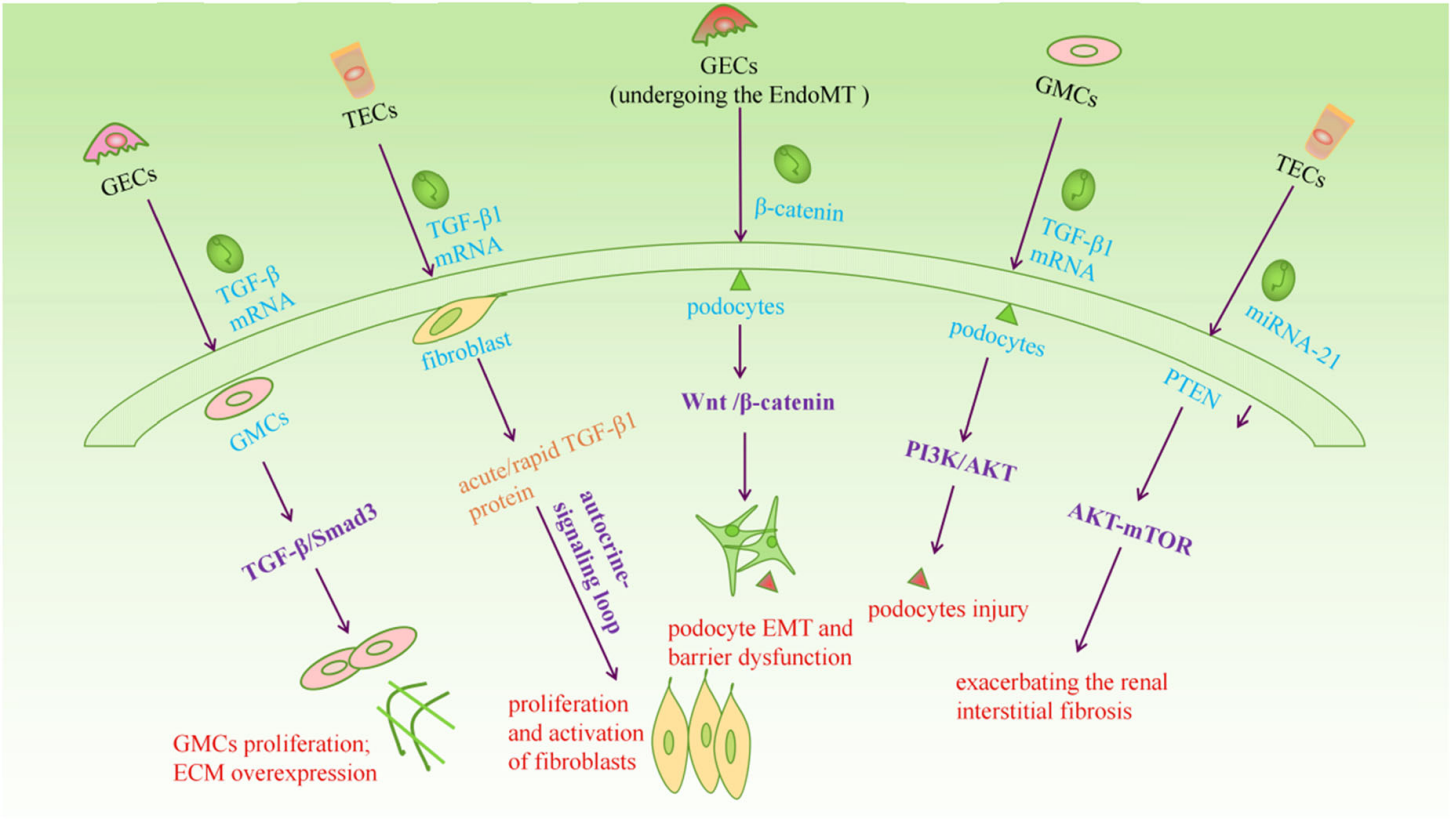
EVs involved signaling pathways involved in renal fibrosis. Injured kidney cells secrete electric vehicles, and transfer their microRNA to target cells, promoting the activation of target cell signaling pathways, leading to changes in the physiological state of target cells and promoting renal fibrosis

**Fig. 4:**
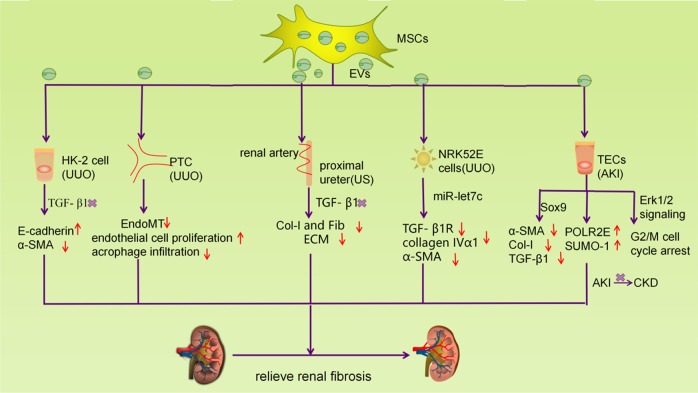
EVs secreted by MSCs reduce renal fibrosis. EVs secreted by MSCs can transfer its contents to target cells, thereby reducing the expression of fibrosis genes in damaged renal cells and ultimately improving renal fibrosis. There are summarizes several classical approaches in the progress

**Table 2:** Advantages and disadvantages of three common EVs separation methods

Method	Advantages	Disadvantages
Ultracentrifugation	Low cost;simple operation	Time-consuming; easy to be polluted; low yield
Immunoisolation	More specific in capturing a small amount of plasma EVs	Quite expensive;not suitable for large-scale;
Ultrafiltration	A small amount of sample can also get enough quantity	Cause some vesicles to deform or rupture

### EVs in renal fibrosis diseases

Renal fibrosis is an inevitable pathological process in which all types of CKD progress to end-stage nephropathy^37^. Renal fibrosis can occur in a variety of common kidney diseases. For example, polycystic kidney disease (PKD) is characterized by the continuous accumulation of cysts caused by renal tubular epithelial cells (RTECs), leading to renal parenchymal damage and non-cystic renal tissue fibrosis^38^. In addition, other common diseases, including DN, LN, and IgAN, can lead to renal fibrosis^38–43^. Several studies have suggested that EVs may be mediators of inflammation, immunosuppression or growth and regeneration. In the kidney, they can originate from blood cells, endothelial cells, podocytes, or tubular epithelial cells, and can be detected in circulation, urine, or inflammation. Here, we describe the contribution of EVs to various renal fibrosis diseases.

**Table 3:** Effect of miRNAs in EVs on renal fibrosis

Injured cells/Urine of kidney disease patient	miRNAs in EVs	Proven	Target protein	Outcomes
High glucose-treated HK-2 cells	miRNA-192 ↑	yes	GLP1R ↓	Renal cell autophagy and apoptosis
Obstructive tubular cells	miR-21 ↑	yes	PTEN ↓	Tubular phenotype transition
Type 2 DN patients (urine)	miR-320c ↑	no	BMP6 ↓	Hk-2 cell injury
Type 2 DN patients (urine)	miR-34a ↑	no	GAS1 ↓	Mesangial proliferation and glomerular hypertrophy
HSA treated HK 2 cells/DKD patients with macroalbuminuria (urine)	miR-4756 ↑	no	Sestrin 2 ↓	TECs EMT and endoplasmic reticulum stress

**Table 4:** Summary of pathological candidate EV biomarkers for common renal disease

Renal diseases	Biomarkers	Model	Type of EVs
Type1 DN	miR-145 WT-1	Mice; human human	Exosomes Exosomes
Type2 DN	miR-15b; miR-34a;miR-636 miR-192 miR-320c	Human Human Human	Exosomes EVs Exosomes
PKD	AGS3	Human	Exosomes
LN	miR-29c miR-26a let-7a;miR-21 (during disease flare)	Human Mouse Human	Exosomes; MVs Exosomes Exosomes
IgA	miR-29c; miR-205 miR-146a α-1-antitrypsin; ceruloplasmin	Human Human Human	Exosomes Exosomes Exosome

## EVs in cellular and molecular mechanisms of renal fibrosis

Numerous studies have shown that EVs are supposed to be potentially active participants in renal fibrosis^24,44,45^. However, the exact mechanism of EVs promoting renal fibrosis remains immature. Further research is needed to determine how EVs are involved in renal fibrosis. Next, we briefly summarize of the existing role of EVs in renal fibrosis at the cellular and molecular levels.

### Factors affecting EVs release during renal fibrosis

Various stress conditions, such as hypoxia^46,47^, acidic pH^48^, and oxidative stress^49^, can increase the secretion of EVs and cause significant changes in the composition of EVs^29,50^. For instance, high glucose stimulates glomerular endothelial cells to secrete exosomes, thereby activating glomerular mesangial cells and promoting renal interstitial fibrosis^51^. A high glucose environment also induces GEC endothelial-mesenchymal transition (EndoMT), which can cause podocyte epithelial-mesenchymal transition (EMT) and dysfunction by releasing exosomes in cells^52^. Moreover, the number of exosomes released by mesangial cells was significantly reduced after high glucose exposure, while the level of miR-145 was increased^53^.

There are other factors that affect the release of the EVs such as renin-angiotensin-aldosterone system (RAAS), vasopressin and uremic toxin. RAAS alters the expression of urinary exosome proteins, with a significant increase in γENaC peptide and urinary proteases^54^. Vasopressin activates V2 receptors in the main cells of renal collecting ducts to stimulate endocytosis of exosomes^55^. In addition, Experiments have shown that uremic toxin including indoxyl sulfate and p-cresyl sulfate directly induces the release of EMPs in vitro^56,57^.

### EVs in cellular mechanisms of renal fibrosis

In the initial stage of renal fibrosis, stimuli lead to an inflammatory response, which involves recruitment of inflammatory cells, release of inflammatory cytokines, and activation of fibrogenic effector cells. All of these results eventually lead to the deposition of extracellular matrix, which is an important mechanism of renal fibrosis^58,59^. Furthermore, EVs also mediate communication among various types of cells, related to renal fibrosis progression^60^. We next clarify how EVs promote renal fibrosis at the cellular level from the following aspects (Fig. 2).

#### EVs in the recruitment of inflammatory cells

The aggregation of inflammatory cells that mediate exosomes is an important cellular mechanism of renal fibrosis^59^. In previous research, it was proved experimentally that MPs from monocytes and endothelial cells induce the secretion of the cytokine chemokine MCP-1 and the cytokine IL-6, which can lead to glomerular inflammation. It also confirmed that monocyte MPs lead to increased podocyte secretion of vascular endothelial growth factor (VEGF), which can affect glomerular permeability in vivo^61^. Recently, research has found that increased release of exosomes transferred CCL2 mRNA from tubular epithelial cells (TECs) in proteinuria renal disease and then delivered them to mesenchymal macrophages, inducing their activation and autocrine recruitment of other myeloid cells^62^. In addition, platelet-derived EV levels are increased in SLE, and the levels of immunoglobulins and complements in EVs are also increased^63^, while the EV components of SLE have also been detected in glomerular deposits in LN patients^64^, suggesting that EVs might contribute to the deposition of immune deposits in glomerular deposits.

Furthermore, current knowledge holds that vascular inflammation is also a common cause of renal interstitial fibrosis, such as anti-neutrophil cytoplasmic antibody (ANCA) vasculitis manifesting as glomerular acute necrotizing vasculitis^65^. ANCA stimulates the release of neutrophil MPs, and increased expression of CD54, IL-6, and IL-8 can be induced by human venous endothelial cells (HUVECs) in vitro, suggesting that EVs can promote inflammation of the vessel wall^66^.

#### EVs in the activation of fibrogenic effector cells

Fibrogenic effector cells are derived from mesenchymal cells, including fibroblasts and myofibroblasts. Fibroblasts are derived from EMT. The release of cytokines also activates fibrogenic effector cells and leads to the deposition of extracellular matrix^59^. Studies have shown that EVs can play a role in activating fibroblast cells. Under hypoxic conditions, damaged tubular epithelial cells produce exosomes containing TGF-β1 and release them to promote the proliferation of adjacent fibroblasts, which manifest the production of α-smooth muscle actin and collagen I^67^. In addition, researches have confirmed that the release of miRNA-23a-rich exosomes is derived from hypoxic TECs that activate macrophages to promote tubulointerstitial inflammation.

GECs undergoing EndoMT can cause podocyte EMT by releasing exosomes^52^, and the role of EMT in renal fibrosis has been a popular topic in recent years^68–71^. Emerging research has confirmed that podocyte MPs induce pro-fibrotic responses in proximal tubule epithelial cells characterized by upregulation of fibronectin and collagen IV expression^72^.

In the progression of fibrosis, there is a TG2 secretion pathway, which is driven by vesicular transport. Subsequently, the secreted TG2 interacts with the protein network responsible for ECM dynamics, leading to fibrosis remodeling and expansion^73^. Moreover, a study found elevated levels of ADAM10 in the urinary vesicles of patients with glomerular nephropathy^74^. Other studies have confirmed that overexpressed exogenous ADAM10 leads to E-cadherin loss and increases α-SMA in HK-2 cells. Although these findings indicate that ADAM10 might be involved in renal tubular epithelial EMT and renal fibrosis^75^, the exact link between them still requires further proof.

#### EVs in damage to resident renal cells

Renal inherent cells mainly include podocytes, mesangial cells, and tubular epithelial cells. The damage to the cells in the process of renal fibrosis development also plays a considerable role^76^. The role of EVs in kidney inherent cell damage has also been confirmed. Injured kidney tubular epithelial cells can affect normal cells and other normal kidney cells by the release of EVs, leading to a vicious circle of renal fibrosis^77,78^. Exosomes from high glucose-treated GECs induce podocyte EMT and barrier dysfunction^52^. In high glucose environments, exosomes derived from GMCs can harm podocytes by inducing apoptosis and inhibiting the expression of cell adhesion membrane protein and wt-1, also suggesting that exosomes can regulate the crosstalk between GMCs and podocytes^79^. Another proof was that the expression of hypoxia-inducible factors in HK-2 cells was upregulated by MPs released by vascular endothelial cells^80^.

Recent research has revealed a new manner of EVs in damage to resident renal cells. Increased platelet MPs in the blood of diabetic patients induce reactive oxygen species production, lower nitric oxide levels, inhibit endothelial nitric oxide synthase and SOD activity, and then increase the permeability of the glomerular endothelial barrier and reduce endothelial thickness. The effect eventually leads to glomerular endothelial function and structural damage, increased permeability, urinary albumin leakage, and DN progression^81^.

### EVs in the molecular mechanisms of renal fibrosis

The molecular mechanisms of renal fibrosis are quite complex and expansive^82^. Various renal diseases develop into renal fibrosis through complicated signaling pathways (Fig. 3)^83^. However, the direct involvement of EVs in the signaling pathway of renal fibrosis is very rare. Furthermore, micro-RNA (miRNA) has been a popular research topic in recent years, and it was proved to be involved in renal fibrosis^84^. Many researchers have indicated that miRNA in EVs could directly or indirectly promote renal fibrosis^85^. Therefore, we illuminate EVs in the molecular mechanisms of renal fibrosis from the above two aspects in this section.

#### Effect of signaling pathway of EVs on renal fibrosis

##### TGF-β signaling pathway

An ocean of evidence has shown that TGF-β/Smad plays an important role in renal fibrosis and is recognized as the main fibrotic factor^86,87^. Recently, it was experimentally demonstrated that TGF-β1 mRNA from glomerular endothelial cell exosomes could mediate GMC activation. Researchers detected that exosome-treated GMCs released by high glucose-treated GECs increased phosphorylated Smad3. That demonstrated exosome-induced GMCs activation dependent on the TGF-β1/Smad signaling pathway^51^. Moreover, mesangial cell phenotype changes induce cell proliferation and activation of fibroblasts, leading to renal fibrosis^88^. In hypoxic conditions, exosomes released by damaged TECs can transfer TGF-β1 mRNA to fibroblasts and transform it into acute/rapid TGF-β1 protein, initiating an autocrine-signaling loop and ultimately leading to the proliferation and activation of fibroblasts^67,89^.

High glucose environments induce an increase in TGF-β1, while TGF-β1 increased the expression of miR-145 in mesangial cells and vascular smooth muscle cells via the Smad pathway, in turn resulting in an increase in miR-145 in exosomes^53^. Prior research has shown that miR-145 can promote vascular muscle cell phenotypes from proliferation to contraction changes. Therefore, it was concluded that increased miR-145 in the exosomes perhaps promote mesangial cell hypertrophy and cytoskeletal remodeling, mediated by the TGF-β1 signaling pathway^53,90,91^. In summary, EVs-mediated TGF-β signaling pathway is currently recognized as a molecular mechanism.

##### Other signaling pathways

Recently, remarkable progress has been made in studying Wnt/β-catenin signaling in the pathogenesis of various renal fibrosis diseases^92,93^. Among these signals, EVs mediated Wnt/β-catenin is worth considering. Recent studies have also confirmed that exosomes derived from cells undergoing EMT increase expression of β-catenin and significantly lead to β-catenin undergoing nuclear translocation, indicating the activation of canonical Wnt/β-catenin signaling^52^. Currently, the evidence of renal fibrosis via EVs mediating the Wnt/β-Catenin signaling pathway is limited, but it is a valuable research direction.

AKT-mediated signaling pathways mediating renal fibrosis disease deserves special attention in the field of exosomes. For example, EMT in renal TECs and renal fibrosis caused by hypoxia are closely related to the activation of PI_3_K/AKT^94^, which has been demonstrated to be an important functional pathway in podocyte injury and renal fibrosis^95^. For example, researchers have shown that the exosomes released from high glucose-induced GMCs can activate the PI_3_K/AKT signaling pathway in podocytes through TGF-β1^79^. In addition, recent proofs have also indicated that increased miRNA-21 levels in MVs secreted by tubule cells activate the PTEN/AKT signaling pathway and aggravates renal interstitial fibrosis^96^. However, the exact function and relationship in the molecular mechanisms of renal fibrosis between EVs and these signal pathways remain to be explored.

#### Effect of miRNAs in EVs on renal fibrosis

MiRNAs are short non-coding RNA species that regulate important functions in cellular events, such as proliferation, differentiation, and immune responses, as well as gene regulation associated with human disease^8^. Specific expressed miRNAs in the kidney act as effectors of TGF-β1 in CKD^97,98^, so their role in renal fibrosis has also been increasingly explored^99–101^. Furthermore, EVs contain miRNAs, mRNAs, proteins and other information materials^102–104^, which have been shown to significantly alter the biological pathways of renal fibrosis disease (Table 3)^32,105^.

MiR-21 has undoubtedly undergone the most in-depth studies in this field. Previous studies have shown that miR-21-mediated MV transport in TECs could have new effects on the mechanism of advanced renal fibrosis^85^. Zhou et al. excluded the effect of pro-fibrosis factors, and according to the molecules that mediate intercellular communication, such communication should be stable and have the ability to regulate genes, suggesting that the above molecules may be miRNAs^85^. In addition, the level of miR-21 in MVs isolated from the urine of UUO mice was significantly higher than that of the control group, and it was also difficult to detect miR-21 in the MV-free urine of UUO mice. Hence, it was concluded that miR-21 in damaged tubule cells is packaged as MVs and passed to normal cells, causing subsequent fibrosis^85^.

There were other miRNAs involved in the mechanism of EVs mediating renal fibrosis, such as miR-192, miR-320, miR-34, etc. Studies have shown that miR-192 in EVs produced by high glucose-treated cells could induce renal fibrosis^77^. The combined analysis of urine exosomes-derived expression levels of miR-192 and TGF-β1 provides new insights into the pathology of early DKD^106^. Moreover, researchers have shown that strong upregulation of miR-320c in the urinary exosomes of patients with type 2 DN and the upregulation of miR-320c expression could lead to downregulation of BMP6^107^, which could, in turn, improve the damage to HK-2 cells induced by TGF-β1^108^. Therefore, it was proposed that the increase in miR-320c in EVs could promote renal fibrosis. Furthermore, miR-34a was upregulated in the urinary exosomes of type 2 DN^109^, and some studies have indicated that miR-34a plays a role in regulating mesangial proliferation and glomerular hypertrophy by targeting growth arrest-specific 1 (GAS1)^110^. Previous studies have shown that proteinuria promotes the progression of DN^111,112^. A possible mechanism was revealed recently. Endoplasmic reticulum (ER) stress and EMT are thought to play key roles in tubulointerstitial fibrosis^113,114^. MiR-4756 could induce HK-2 cell damage by promoting EMT and ER stress, and the expression of miR-4756 in EVs from HAS-treated HK-2 cells was increased^115^.

## Clinical application of EVs in renal fibrosis disease

In view of the natural characteristics of genetic information transfer, the possibility of using EVs for therapeutic purposes is currently being studied. Firstly, the molecular content of EVs is like the fingerprint of its primordial cells. The goods of EVs vary with the state of the disease, and EV is positioned as a potential source of discovering new disease biomarkers. Urine EVs is a good diagnostic material because it is easy to collect and reflect the pathophysiological status of the kidney. They may replace kidney biopsies in the future. Secondly, stem cell-derived EVs seem to naturally mediate tissue regeneration under certain conditions. Here we summarize the latest advances in the potential application of stem cell-derived EVs in renal diseases. Third, recent evidence suggests that EVs can be used as drug delivery vectors to treat and target specific cell types. Therefore, EVs emerge as an effective genetic information transfer agent, which supports a series of biological processes and has therapeutic potential.

### EVs as potential biomarkers in renal fibrosis

EVs have been found to be novel, non-invasive markers that hold the promise of being tools for mechanical research, including disease progression and the possible monitoring of therapeutic effects^116^. Recent studies have found that the RNAs and proteins contained in EVs not only reflect the biological information of the mother cells, but they also reflect their physiological and pathological status, related to the occurrence and development of renal fibrosis (Table 4)^117^.

#### DN

As the most common CKD in western countries^118^, approximately 20–40% of diabetic patients eventually develop diabetic kidney disease (DKD)^119^. Today, the function of EVs in the diagnosis of DN has been extensively studied. MiRNAs in EVs are most commonly used to diagnose early DN. It was reported that urinary exosomal miRNA levels were altered in patients with type 1 DN, and miR-145 in urinary exosomes could be a new candidate biomarker^53^. Studies have also shown that miRNA-192 in urinary exosomes could be used to diagnose early DN^120^. Upregulation of miR-15b, miR-34a, and miR-636 in urinary exosomes was also found in patients with type 2 DN^109^. There have also been experiments showing that upregulation of miR-320c in urinary exosomes might be a new potential marker for the progression of type 2 DN disease^107^. In addition, there are distinct differences in the levels of miRNA-215 and miRNA-494 in diabetic rats with severe kidney injury or high glomerular sclerosis, compared to diabetic rats with only moderate pathology^121^.

In addition to miRNAs, the level of Wilms tumor 1 (WT1) also changes in the urinary exosomes of patients with DN, which might reflect potential damage^122^. Moreover, WT1 mRNA levels reflect the damage to the diabetic glomeruli in podocyte-derived signal transduction factors (PDSTFs) from urinary exosomes, which in exosomes can predict the decline of eGFR in patients with DN over the next few years^123^. Furthermore, studies have demonstrated that urinary podocyte MPs are early and sensitive markers of diabetic podocyte/glomerular injury^124,125^.

#### Other kidney diseases

As far as we know, other common renal fibrosis diseases include PKD, LN, and IgA. The urine exosomal PC1/TMEM2 or PC2/TMEM2 ratio could have utility in the diagnosis and monitoring of PKD^126^. Recently, experiments showed for the first time that there is a significant difference in the expression of urinary-exosomal activator of G-protein signaling 3 (AGS3) between PKD patients and healthy individuals. Therefore, AGS3 in urinary exosomes was considered to be a good biomarker for PKD^127^. Moreover, studies have shown that miR-29C in urinary exosomes could be used as novel, non-invasive markers of LN progression^128,129^. At the same time, studies have confirmed that the level of miR-26a in the urinary exosomes of LN patients is significantly higher than that in healthy groups^130^. Recently, significant downregulation of let-7a and miR-21 in the urinary exosomes of active LN patients was also confirmed^131^. Furthermore, experiments also confirmed that, compared with a healthy control group, the expression of miR-29c and miR-205 in urinary exosomes in IgAN patients was significantly down-regulated, while miR-146a was significantly upregulated^132^. In addition, two proteins, α-1-antitrypsin (Serpina1) and ceruloplasmin (CP), could act as biomarkers of IgAN because they are increased in urinary exosomes^133^.

### Application of stem cell-derived EVs in renal fibrosis

In recent years, there has been an unprecedented increase in research on improving renal fibrosis by stem cell-derived EVs. A growing body of evidence supports the impact of mesenchymal stem cells (MSCs) on repair fibrosis in ureteral obstruction by releasing EVs (Fig. 4)^23,134^. Unilateral ureteral obstruction (UUO) is a classic model for studying renal parenchymal inflammation and fibrosis^135^. EVs derived from MSCs can alleviate renal tubular injury and fibrosis at 2 weeks after UUO and improve renal function, and EVs also reverse morphological changes induced by TGF-β1, resulting in upregulation of E-cadherin expression and decreased α-SMA secretion in HK2 cells^136^. Similarly, MPs derived from kidney-derived MSCs reduce endothelial cell-to-mesenchymal transition, promote endothelial cell proliferation, and inhibit inflammatory macrophage infiltration, further reducing renal fibrosis in mice within 7 days after UUO^137^. In another study, a UUO mouse experimental model was used to demonstrate that MSCs exogenously transferred miR-let7c to the injured kidney through exosomes, resulting in up-regulation of miR-let7c and reduction of collagen IVα1, α-SMA, and TGF-βR1 expression, ultimately improving kidney structure^138^. These studies confirmed the important anti-fibrotic and renal protective effects of MSCs in obstructive nephropathy (ON).

Moreover, for AKI induced late fibrosis, adipose-derived mesenchymal stem-derived exosomes upregulate the expression of renal tubular SOX9, promote tubular regeneration, attenuate ischemia-induced AKI, and reduce subsequent renal fibrosis^139^. It was also found that exosomes derived from MSCs could improve the apoptosis of renal tubule cells induced by cisplatin and promote the recovery of renal tubular function and morphology^140^. Furthermore, MVs derived from human adult mesenchymal stem cells could play a renal protective role by inhibiting apoptosis of renal tubular epithelial cells and promoting their proliferation^141^.

### EVs as a drug delivery

EVs, as a biologically active system for substance transfer between cells, have great potential as therapeutic drug carriers. In addition, they can be used to deliver specific substances or to improve their uptake capacity through engineering^142^. Although current studies have proved that EVs can be used as delivery vectors for therapeutic drugs^143^, there are still many challenges to overcome before they can be directly applied in clinical practice. Since the properties of EVs are directly related to the conditions under which they are produced and the cells that produce them, it is essential to establish the characteristics of EVs from different sources for the repeatability and safety of subsequent applications. Establishing a large number of methods for preparing EVs is the precondition for clinical trials of EVs. Currently, there are few studies on EVs as a drug delivery in renal fibrosis, but there is no doubt that this is a promising research direction.

## Opinions on open questions

Our opinions on open questions are as follows. In view of the first problem, the current research is more believed that in vivo, proteins on the surface of EVs can be recognized by receptors of distant cells, thus inducing signal transduction similar to intercellular communication. Other proteins, such as enzymes or transcription factors, can be absorbed by cells and play a role in target cells. The second question is about the discussion of the main components that mediate the functions of EVs. According to a large number of studies, EVs can transmit the genetic information from donor cell to recipient cell. Therefore, the content of EVs has attracted the attention of researchers. Current studies have found that the contents of EVs may include RNAs, DNAs, and proteins. However, after reading a lot of literature, we found that most researchers focused on RNAs in EVs-mediated function. We believe that with the improvement of isolation methods and the increasing understanding of EVs, proteins, DNAs and even other undetected contents in EVs may be excavated in terms of function mediation. For the third issue, we analyze the current researches and believe that it is promising to focus more attention on RNAs in EVs in renal fibrosis. Undoubtedly, a large number of studies have confirmed that RNAs in EVs plays an important role in renal fibrosis. Based on a large number of experimental results, we can say that it is reasonable to pay attention to RNA in EVs. However, with the progress of the times and the maturity of research methods, we believe that in renal fibrosis, more eyes will be transferred to other EVs contents.

## Conclusion

EVs, characterized by dynamic, stable carrying of biological information, are expected to be a very good biomarker for lesion degree or targeted therapeutic vectors for renal fibrosis disease. Although the prospects are very promising, we still have an arduous and lengthy road to travel to realize this potential. The contributions of EVs to normal kidney physiology and their ability to regulate pathophysiological processes remain to be confirmed. We still do not know the action mechanism of EVs and how to manipulate them effectively. How to obtain exosomes on a large scale for clinical treatment will also be a focus of future studies. Regardless, the function of EVs, and their changes to the quality and quantity in renal fibrosis diseases are being increasingly understood with a now rapidly expanding body of evidence. Through further in-depth research into the roles of EVs in the development of renal fibrosis, we will provide a more theoretical basis and additional intervention targets for anti-fibrotic therapy. In short, the challenge persists, requiring us to explore more in this field.
